# Where We Are and Where We Are Going in Nut Research

**DOI:** 10.3390/nu15071691

**Published:** 2023-03-30

**Authors:** Jordi Salas-Salvadó, Stephanie K. Nishi, Joan Sabaté, Emilio Ros

**Affiliations:** 1 Universitat Rovira i Virgili, Departament de Bioqímica i Biotecnologia, Alimentació, Nutrició, Desenvolupament i Salut Mental (ANUT-DSM), 43201 Reus, Spain; 2 Institut d´Investigación Sanitària Pere Virgili (IISPV), 43201 Reus, Spain; 3 Centro de Investigació Biomédica en Red Fisiopatología de la Obesidad y la Nutrición (CIBEROBN), Institute of Health Carlos III, 28029 Madrid, Spain; 4 Toronto 3D (Diet, Digestive Tract and Disease) Knowledge Synthesis and Clinical Trials Unit, Toronto, ON M5C 2T2, Canada; 5 Clinical Nutrition and Risk Factor Modification Centre, St. Michael´s Hospital, Unity Health Toronto, Toronto, ON M5C 2T2, Canada; 6 Center for Nutrition, Lifestyle and Disease Prevention, School of Public Health, Loma Linda University, Loma Linda, CA 92350, USA; 7 Lipid Clinic, Endocrinology and Nutrition Service and Institute d´Investigacions Biomèdique August Pi Sunyer, Hospital Clínic, 08036 Barcelona, Spain; 8 Institut Dínvestigació Sanitària Pere Virgili (IISPV), 43201 Reus, Spain

Nuts have been part of the human diet for thousands of years [1]. Traditionally, nuts have been incorporated as an ingredient in many dishes, and over the years, nuts have been consumed in various forms from raw or minimally processed to more processed forms and eaten as snacks as well as included within recipes for main dishes.

In the last decades of the 20th century, the prevailing belief that dietary fat was harmful was at the basis of nuts being discouraged due to their high fat content. However, this perspective started to change following the first scientific studies demonstrating the potential health benefits of nut consumption. In 1992–1993, seminal publications from Loma Linda University showed that walnut consumption significantly reduced serum cholesterol [2] and that the frequency of nut consumption was inversely associated with coronary heart disease incidence according to data from the Adventist Health Study cohort [3]. Since then, many randomized clinical trials, epidemiological studies, and in vitro/in vivo mechanistic studies have explored and described the role of the consumption of different types of nuts on reduced incidence of cardiovascular disease and all-cause mortality, management of lipid disorders, and glycaemic control, without undue effects on body weight or overall adiposity, among other cardiometabolic and health-related risk factors and conditions [4]. More recently, several studies have examined the potential beneficial effects of nuts on the gastrointestinal system, cognitive performance, fertility, and different types of cancer, as well as the potential mechanisms implicated in the observed benefits. Importantly, landmark studies, such as the Adventist Health Study, the Nurses’ Health Study, the Health Professionals Follow-up Study, the Physicians’ Health Study, and the PREDIMED trial, have consistently reported that frequent nut consumption was associated with a lower risk of different cardiovascular outcomes [4] and all-cause mortality.

Based on the available scientific evidence, specific health claims have been accepted for nuts. Particularly, the Food and Drug Administration (FDA) has authorized qualified health claims for nuts in general and for walnuts and macadamias in particular concerning heart disease prevention when daily consuming one and one-half oz (42 g). However, the European Food Safety Authority (EFSA) has only agreed on a specific health claim for walnuts regarding beneficial effects on endothelial function. At the same time, nuts have been recommended over the last two decades by several health organizations and agencies worldwide.

Due to the accumulating evidence on nut consumption and health outcomes, we thought it would be important to recapitulate and examine in detail what is well known and established, and what avenues of knowledge are still lacking in nut research. It is for this reason that we organized the NUTS 2022 Conference with the slogan: *Where we are and where we are going in nut research*.

The NUTS 2022 Conference offered the unique opportunity to bring together experts in the field of nut research from around the world with the following aims: (a) to summarize all the evidence related to the beneficial effects of nuts on health; (b) to identify new topics, needs, and opportunities in nut research; (c) to share knowledge with food industry and set new primary objectives for the future; and (d) to develop these scientific proceedings summarizing the current knowledge and new opportunities of research in the nut–health axis.

We believed it would be important and extremely useful to summarize and discuss future lines of nut research in the context of a multidisciplinary group of investigators with expertise in different fields for the benefit of: (1) the investigators, since it allows us to interact, share new ideas, and establish collaborations in the future; (2) the food industry, because they need to know that we know relatively little and that knowledge needs to be invested in; and (3) health agencies, because they need the most up-to-date knowledge to establish appropriate public health recommendations.

The NUTS 2022 Conference took place on 20–21 October 2022, and was organized in Reus by the University Rovira i Virgili together with Institut d’Investigació Sanitària Pere i Virgili (IISPV) and the Ciber Fisiopatología de la Obesidad y Nutrición (CIBEROBN) of Instituto de Salud Carlos III of Spain.
